# Antidepressant Drugs for Seizures and Epilepsy: Where do we Stand?

**DOI:** 10.2174/1570159X20666220627160048

**Published:** 2023-06-15

**Authors:** Martina Tallarico, Maria Pisano, Antonio Leo, Emilio Russo, Rita Citraro, Giovambattista De Sarro

**Affiliations:** 1 System and Applied Pharmacology, Science of Health Department, School of Medicine, Magna Graecia University of Catanzaro, Catanzaro, Italy

**Keywords:** Depression, antidepressant drugs (ADs), animal models, clinical studies, seizures, epilepsy

## Abstract

People with epilepsy (PWE) are more likely to develop depression and both these complex chronic diseases greatly affect health-related quality of life (QOL). This comorbidity contributes to the deterioration of the QOL further than increasing the severity of epilepsy worsening prognosis. Strong scientific evidence suggests the presence of shared pathogenic mechanisms. The correct identification and management of these factors are crucial in order to improve patients’ QOL. This review article discusses recent original research on the most common pathogenic mechanisms of depression in PWE and highlights the effects of antidepressant drugs (ADs) against seizures in PWE and animal models of seizures and epilepsy. Newer ADs, such as selective serotonin reuptake inhibitors (SRRI) or serotonin-noradrenaline reuptake inhibitors (SNRI), particularly sertraline, citalopram, mirtazapine, reboxetine, paroxetine, fluoxetine, escitalopram, fluvoxamine, venlafaxine, duloxetine may lead to improvements in epilepsy severity whereas the use of older tricyclic antidepressant (TCAs) can increase the occurrence of seizures. Most of the data demonstrate the acute effects of ADs in animal models of epilepsy while there is a limited number of studies about the chronic antidepressant effects in epilepsy and epileptogenesis or on clinical efficacy. Much longer treatments are needed in order to validate the effectiveness of these new alternatives in the treatment and the development of epilepsy, while further clinical studies with appropriate protocols are warranted in order to understand the real potential contribution of these drugs in the management of PWE (besides their effects on mood).

## 1 INTRODUCTION

Epilepsy, a disorder of brain function characterized by a lasting predisposition to generate periodic unprovoked seizures, is one of the most common and debilitating disorders of the central nervous system (CNS) with incidence rates, ranging between 40 and 70/100, 000/year worldwide [1]. It is the second most burdensome neurologic disorder in terms of disability-adjusted life years [2]. Current therapy is symptomatic: available antiseizure medications (ASMs) inhibit seizures but neither of these prevents the development nor progression of epilepsy (epileptogenesis). For a long time, epilepsy has been defined as a disorder of the CNS manifested only by the occurrence of epileptic seizures, and its treatment was focused on the achievement of complete seizure control without significant side effects from treatment. Over the past decade, however, there has been increasing recognition that spontaneous recurrent seizures (SRSs) are only the clinical manifestations of a more complex disorder. In fact, for many patients, epilepsy also includes a number of psychiatric and neurological comorbidities (depression, anxiety, autism spectrum disorders, sleep disorders, attention deficits, cognitive impairment, and migraine), which can precede or follow the onset of epilepsy and have a profound negative impact on the patient’s QOL among other factors [3]. Roughly 50% of adults with active epilepsy present at least one comorbid medical condition [4]. The need to include the comorbidities in the management of patients with epilepsy (PWE) stems from their negative impact on QOL, morbidity, and mortality, and from the prevalence rates which is up to eight times higher in people with epilepsy than in the general population [5]. For these reasons, the new definition of epilepsy proposed by a task force of the International League against Epilepsy (ILAE) implies a need to factor-in the existence of comorbid conditions. The comorbidities are integral not only to classification but also to the diagnosis of epilepsy, given their epidemiological and socioeconomic weight and the impact on prognosis and QOL of PWE [3]. Furthermore, comorbidities are frequently more harmful to patients than ongoing seizures, significantly affecting the lives of these patients, increasing the suicidal risk [6], and decreasing the tolerability of ASMs [7]. Therefore, the primary goal of epilepsy treatment is not only restricted to the achievement of complete seizure control but must also include the management of neurological, psychiatric, and cognitive comorbidities. Among the co-morbidities associated with epilepsy, psychiatric conditions (*e.g.*, depression, anxiety disorder, and psychosis) are among the most common and problematic, and the correct choice of drugs is very important in the treatment of these patients. Moreover, it has been suggested and widely accepted that epilepsy and its comorbidities have a bidirectional relationship; for example, depression increases the risk for epilepsy, and epilepsy, the risk for depression likely sharing a common underlying pathogenesis [8]. This complex bidirectional relationship between epilepsy and depression in terms of cellular and molecular processes, including neurotransmitters, neuroinflammation, aberrant neurogenesis, and hypothalamic pituitary adrenal (HPA) axis dysregulation, requires a combined therapeutic approach. This review summarizes the recent data on the effects of Ads on epilepsy in experimental animal models and in patients, and the common pathological mechanisms that have been identified as potential substrates for depression and epilepsy [8].

## 2 EPILEPSY AND DEPRESSION COMORBIDITY

Depression is likely the most common psychiatric comorbidity in adults and pediatric PWE, with a prevalence rate estimated between 22 and 23% for depression established in all types of epilepsy. Depression in PWE is termed, by Diagnostic and Statistical Manual of Mental Disorders fifth edition (DSM-5), as a depressive disorder due to other medical conditions [9]. Depression is a broad and heterogeneous term that may include different types of conditions. In general, depressive disorders are characterized by complex symptomatology including low mood, diminished interest in normal activities, insomnia or increased sleep, mental slowing and poor concentration, significant weight loss or gain and activity patterns, psychomotor agitation or retardation, decreased energy and libido, and suicidal ideation. Depression has a negative impact on the QOL in PWE acting independently even on seizure control itself [3]. As evidence from epidemiological studies, 1 in 5 PWE have depression and are twice as likely as people without epilepsy to experience these disorders during their lifetime [10]. Similar incidence rates have been identified in children and adolescents with epilepsy and, as shown in adults, the prevalence rate in children seems to reflect the severity of the underlying seizure disorder [11]. On the other hand, people with depression have a seven-fold increase in the risk of developing epilepsy [12]. In particular, in those with medically resistant epilepsies, the incidence and prevalence are higher than in the general population [13]. Depression is a greater predictor of impaired QOL than epilepsy-related variables, such as illness duration or seizure frequency [14]. In addition to debilitating conditions, PWE and different types of clinical depression (*e.g.* dysthymia and MDD) reported higher levels of perceived seizure severity, recovery time, and disability than individuals with epilepsy without depression [15]. Therefore epilepsy and depression are bidirectionally linked, with depression increasing the risk for epilepsy and epilepsy increasing the risk for depression [8]. Although depression has been repeatedly found to have an adverse impact on PWE’s QOL often to a greater extent than seizures themselves, it remains unrecognized and/or untreated in a large percentage of patients [16]. This may be due to various factors, including the lack of a systematic approach in the diagnosis and management of psychiatric disorders but also to the increased risk of psychiatric side-effects on both mood and cognition caused by some ASMs [17]. ASMs are well-known to induce psychiatric disorders in PWE including anxiety, depression, irritability, and hallucinations, making their use in people with a history of seizures or psychiatric disorders problematic. These adverse events vary in duration and occur from a few hours to several weeks after the introduction of treatment [17]. This is a major confounding factor that is difficult to control in PWE studies. Similar to human studies, basic research in animal models of seizures and epilepsies has described depression-like behaviors and anhedonia comorbidity. These epilepsy models not only make it possible to study depression-like behavior and common neurobiological mechanisms without the interference of psychosocial and iatrogenic factors that are present in human studies, but also to create new treatments for the co-occurrence of epilepsy and depression. Depression-like behaviors have been described in the generalized spike-wave epilepsy model involving models for human absence epilepsy (WAG/Rij and GAERS rats) [18], models of audiogenic generalized tonic-clonic seizures (GEPRs) [19], models of limbic epilepsy (electrical kindling) [20] as well as models of epileptogenesis following status epilepticus (models of TLE) [21]. In epilepsy, psychiatric disorders, and in particular depressive conditions, have characteristic symptomatology, not easily recognizable in clinical practice. This can occur for post-ictal mood changes, which are less diagnosed by clinicians due to their short duration [9]. Given the heterogeneity of clinical scenarios where psychiatric symptoms occur, it is easy to understand that validated screening instruments such as the DSM-5 and the International Classification of Diseases (ICD) may not be always valid. Therefore, recently, ILAE provided evidence-based recommendations for the treatment of depression in adults with epilepsy [22].

## 3 COMMON PATHOLOGICAL MECHANISMS OF EPILEPSY AND DEPRESSION

The cause of the increased risk of depression in PWE is multifactorial; biological factors such as neurological features of epilepsy, age of onset, type, frequency, and severity of seizures, epilepsy syndrome, anatomical location, and psychosocial and iatrogenic factors (*e.g.*, ASMs, surgery) are considered predictors. Furthermore, the existence of common pathological mechanisms in both epilepsy and psychiatric disorders is widely accepted (*e.g.*, neuroinflammation, aberrant neurogenesis, HPA axis dysregulation, altered tryptophan metabolism genes, traumatic brain injury, early life stress) [23]. Here, we will review the most important or better studied; namely, neuroinflammation, altered monoaminergic transmission and other neurotransmitters, HPA axis dysregulation, and altered neurogenesis.

### 3.1 Neuroinflammation

Inflammation is involved in pathological processes taking place in the epileptic brain being directly associated with seizures and epileptogenesis [24, 25]. On the other hand, neuroinflammation also plays an important role in the pathogenesis of a wide range of neurological conditions including psychiatric disorders such as anxiety and depression, and cognitive impairments [26, 27]. Elevated release of inflammatory mediators such as IL-1β, IL-2, IL-6, and TGF-α as well as increased expression of COX-2/E2 (PGE2), high mobility group box 1 (HMBG1) toll-like receptors 4 (TLR4) released from neurons, astrocytes, and microglia, following epileptogenic insult (SE, TBI) cause development of seizures and possibly depression [28, 29]. The role of cytokines as neuromodulators in the epileptic and depressive brain is based on the results of studies in humans and experimental animals; blood serum and cerebrospinal fluid (CSF) levels of cytokines (IL-1ra, IL-1β, IL-6, IL-17) are increased in PWE [30, 31] whereas increased plasma levels of IL-1β have been specifically connected with epilepsy-associated depression [32]. In patients with TLE, CSF and plasma concentrations of IL-6 are increased in comparison to healthy controls [33, 34]. Similarly, increased levels of plasma IL-1β, IL-6, TNF-α, and CRP have been detected in TLE animal models [35, 36]. Proinflammatory mediators can modulate neuronal excitability *via* mediating inflammation-related interactions between glia and neurons that play a role in reducing seizure threshold [37]. At the same time, neuroinflammation is clearly involved in neuronal death. Immunohistochemical studies on experimental epilepsy models demonstrated that several inflammatory mediators are spontaneously induced by brain injury, or by seizures, in locally activated astrocytes and microglial cells. Induction of inflammatory mediators also can further affect blood-brain barrier permeability, suggesting that inflammation spreads from glia to the brain microvasculature. Furthermore, macrophages and granulocytes also release inflammatory mediators that entering into the brain during epileptogenesis [38]. At the same time, proinflammatory cytokines and acute-phase proteins are found significantly higher in plasma or cerebrospinal fluid (CSF) concentrations among patients with major depressive disorder (MDD), with a fairly unanimous consensus of increases in several interleukins (IL) such as IL-1β, IL-6, tumor necrosis factor-alfa (TNFα), and C-reactive protein (CRP) compared to healthy controls [39, 40]. Furthermore, higher levels of IL-6 predicted over time the chronicity of depression, as well as higher severity of depression at follow-up [41, 42]. Different prospective studies have suggested that plasma levels of CRP may serve as a potential biomarker to predict the onset of MDD [43]. Treatment with SSRIs and SNRIs has shown an anti-inflammatory effect in clinical trials, as they proved to decrease the levels of pro-inflammatory cytokines and to diminish symptoms in a subset of patients with MDD [44, 45] although other previous studies had opposite results [46]. Depression models in rodents are associated with an increase in pro-inflammatory cytokine levels [28]. Forced swimming induces an increase in the levels of pro-inflammatory cytokines IL-1β, and IL-6 in the hippocampus and activation of microglia, in rodents with stress-induced anhedonia [47]. On the other hand, animal models of conditions associated with inflammation are characterized by depressive-like behavior. It is known that “sickness behavior” induced by systemic inflammation is reversible, but after it is resolved, the depressive-like behavior remains [48]. Thus the intricate balance between the physiological and pathological concentrations of the pro- and anti-inflammatory cytokines is causally linked to many of the major symptoms of MDD [49, 50]. Inflammatory mediators can stimulate receptors on neurons and glial cells, thus causing immediate channel modifications, presynaptic changes in neurotransmission, and activation of many signaling pathways. This suggests that inflammation, together with seizures, participates in neuropsychiatric comorbidities of epilepsy [51]. This hypothesis has been confirmed by some studies, that show significant neuroanatomical changes in patients with depression, such as decreased glial/ neuronal cell density in the cingulate gyrus, atrophy of the temporal and frontal areas; changes in the amygdala, hippocampus, entorhinal cortex, and lateral temporal cortex, as well as the prefrontal, orbitofrontal, and medial-frontal areas of the cortex and, to a lesser extent, the thalamus and basal ganglia; functional impairments to the temporal and frontal lobes [52, 53]. Many of these changes are also seen in some cases of temporal lobe epilepsy (TLE), supporting the idea that the same neurobiological changes underlying depression can also lead to the occurrence of seizures and indicating that this common mechanism may become a suitable target for both [54]. Pro-inflammatory cytokines can affect tryptophan metabolism, which is crucial for serotonin synthesis, and as a consequence, disrupt serotoninergic transmission. One pathway involves tryptophan hydroxylation to serotonin whereas another pathway involves the conversion of tryptophan to kynurenine by the action of dioxygenase enzymes (IDO or TDO). Tryptophan availability as a substrate is rate-limiting for both enzymes. Several cytokines have been implicated in the increased synthesis of kynurenine due to the over-activation of IDO or TDO. Elevated levels of IDO and TDO are strongly associated with major depression and depressive-like phenotypes in animal models [55] and may also explain the ineffectiveness of SSRIs in alleviating depression associated with epilepsy [56]. Likewise elevated levels of IDO enzyme activity have been reported in PWE [57]. The kynurenine pathway appears to be operant in both disorders and its pharmacological modulation can be used for the safe treatment of epilepsy and associated depression

### 3.2 Altered Neurogenesis

Aberrant neurogenesis may mediate the development of spontaneous recurrent seizures (SRSs) and associated psychiatric comorbidities following epileptogenic events [58, 59]. SRSs in chronic TLE is associated with significantly reduced neurogenesis, abnormal reorganization of neural networks, and loss of functional inhibition in the hippocampus and extrahippocampal regions [60]. Such changes are also accompanied by learning and memory impairments and depression [59]. Reduced neurogenesis in the hippocampus is considered one of the key mechanisms in the development of MDD [61], so neurogenesis reduction in TLE might be a basis for TLE comorbidity with depression [62].

Disrupted granule cell neurogenesis could also be a potential mechanism underpinning this relationship. Chronic reductions in neurogenesis following stress and depression would reduce the number of normal granule cells present in the dentate gyrus, making the hippocampus more vulnerable to seizure spread. On the other hand, epilepsy is associated with increased production and aberrant integration of new cells early in the disease, and decreased production of new cells late in the disease, further increasing the risk of depression. In both cases, these changes in neurogenesis play important roles [59].

### 3.3 Hypothalamic-Pituitary-Adrenal (HPA) Axis

The hyperactivation of the HPA axis may be related to both depression and epilepsy [63]. Increased activity of the HPA axis is frequently observed in major depression [64] and likely constitutes a common pathway that may also be disturbed in the combination of TLE and depression. Hence, disturbances in HPA axis activity have been implicated as a possible pathogenic mechanism underlying the association between both pathologies. Animal studies have reported HPA axis dysregulation in epilepsy, resulting in elevated circulating glucocorticoid (corticosterone/cortisol) levels; these elevated levels promoted excitatory neurotransmission and potentiated excitotoxicity of hippocampal pyramidal neurons, leading to dendritic atrophy, spine loss, as well as decreased neurogenesis. Elevated basal circulating corticosterone/cortisol levels are well correlated with seizure number and severity [63]. Patients with TLE can exhibit abnormal stress responses which facilitate ictal discharges and increase vulnerability to the development of comorbid psychopathologies [65]. The hyperactivity of the HPA axis negatively affects the structure and function of the brain temporal lobe, an area deeply involved in both TLE and mood disorders; thus in chronic epilepsy, severe neurodegeneration in limbic areas is reported, and thus, the finely tuned regulation of amygdala and hippocampus on HPA axis is disturbed, which may contribute to epileptogenesis and associated depression [23, 66]. The hyperactivity of the HPA axis was shown both in TLE patients with concurrent depression and in a TLE/depression animal model [67, 68]. Several studies in animal models of epilepsy also indicated the role of elevated corticosterone levels in the genesis of epilepsy and comorbid depression. High cortisol levels have been identified as pivotal pathogenic mechanisms of MDD [69]. Early life stress, which is recognized to up-regulate HPA axis responsivity, imparts an enduring vulnerability to experimental limbic epileptogenesis [70]. Additionally, pretreatment with corticosterone induces depression [71, 72] and accelerates the kindling process in rats [73, 74]. Elevated circulating corticosterone levels have been found in pentylenetetrazole (PTZ) kindled animals associated with depression [68, 75]. Long-term corticosterone exposure had also been reported to attenuate serotonin response and alter the functioning of the 5-HT_1A_ receptor functioning in the brain [76]. Similarly to animal models, also in humans, elevated cortisol levels are reported to cause various structural abnormalities in the brain, such as hippocampal atrophy, frontal lobe atrophy, decreased cortical thickness, decreased size of the cingulate gyrus, rostral and caudal orbitofrontal cortices, and the dorsolateral prefrontal cortex are some of the most prominent alterations observed in epileptic people with MDD [77, 78]. A persistent HPA axis hyperactivity has been observed after seizures in PWE, suggesting an impairment of the inhibitory control of the HPA system [65]. Thus, HPA axis dysregulation seem to play an important role both for epilepsy and depression and therefore in their comorbidity [79, 80].

### 3.4 Disturbances in Neurotransmitters

#### 3.4.1 Glutamatergic and GABA Neurotransmissions

Epilepsy and depression also share common receptor and transduction mechanisms in their neurobiological causes [81, 82]. The excitatory/inhibitory imbalance in epilepsy has long been known; glutamate is the excitatory neurotransmitter with a pivotal pathogenic role in the development of epileptic seizures and epileptogenesis [83, 84], while GABAergic inhibition has been traditionally viewed as the main mechanism counterbalancing glutamatergic excitation and preventing hypersynchronous neuronal discharges. Indeed, deficits in GABAergic functions most commonly result in a hyperexcitable epileptic state [85]. However, the involvement of GABA and glutamate have been also recognized in MDD [86]. High glutamate levels have been identified both in animal models of depression and in plasma and cerebrospinal fluid of patients with major depression; this alteration has been correlated with the severity of the depressive disorder [87-90]. Of note, glutamatergic and monoaminergic systems are closely interconnected, as evidenced by the projection of glutamatergic neurons from the cortex to the locus coeruleus, raphe nucleus, and substantia nigra [91]. Hyperglutamatergic neurotransmission in the hippocampus is a central factor in the pathogenesis of epilepsy, depression, and cognitive impairments [92]. It induces neurodegenerative changes, aberrant signaling of glutamatergic receptors, excitotoxicity, and oxidative stress, which also contribute to the comorbidity of TLE and psychiatric diseases [93-95]. Preclinical studies report altered levels of glutamate, synaptic markers, and dendrite formation in rodent models following both acute and chronic stress procedures [96]. Acute stress increases extracellular glutamate in the prefrontal cortex and hippocampus, and this has led to the hypothesis that glutamate-mediated excitotoxicity *via* N-methyl-D-aspartate (NMDA) receptors is responsible for the atrophy of neurons in these brain regions [97]. Chronic stress also decreases the number and function of synapses of pyramidal cells [86, 98]. There is also evidence of NMDA receptor subunit NR1 up-regulation in the molecular layer of the dentate gyrus in unmedicated TLE patients with depressed mood in comparison to TLE patients without psychiatric comorbidities [95]. In the case of GABA (γ-aminobutyric acid type A), a low GABA tone has been documented in CSF [99] and the cortex [100] of patients with major depression compared to control subjects [101, 102]. Studies of transcranial magnetic stimulation also report a reduction in GABA function in depressed patients, determined by analysis of cortical inhibition, which indicates deficits in the function of both GABA_A_ and GABA_B_ receptors [103]. Deficits in GABAergic inhibition may be a contributor to mood disorders and comorbidity in epilepsy [104, 105]. More specifically, it seems that major depression was associated with GABA_B_ receptor deficits, whereas resistant MDD was associated with both GABA_A_ and GABA_B_ deficits, suggesting that more marked GABAergic deficits are associated with more severe symptoms [106, 107]. Preclinical studies of chronic stress also report reductions in levels of GABA synthetic enzymes and neuropeptides in the medial prefrontal cortex and other cortical brain regions [99, 108-110]. Studies in animals and PWE suggest a loss of hippocampal GABA-mediated inhibition underlying neuronal hyperexcitability [111, 112]. Changes in the GABA system associated with TLE may rely on several factors such as comorbid anxiety and depression [104]. It has been reported that ADs, such as serotonin reuptake inhibitors(SSRIs), can influence the GABAergic system [113], and ion channels suggest a possible mechanism by which these drugs could also exert an antiseizure effect.

#### 3.4.2 Monoaminergic Neurotransmission

Monoaminergic neurotransmission (serotonin (5-HT), noradrenaline (NE), and dopamine (DA)) is classically correlated with depressive disorder etiology and are the targets of its pharmacotherapy.

Decreased serotonergic tone represents a pivotal pathogenic mechanism of depression; in pre-clinical and clinical studies, serotonergic signaling deficit (including reductions in serotonin (5-HT) neurons and their projections and increases in 5-HT autoinhibition) has been associated with MDD pathogenesis [114]. Such insufficient 5-HT signaling may result from both reduced release and lower postsynaptic sensitivity as patients with major depression demonstrate both decreased plasma and platelet levels of 5-HT, as well as blunted prefrontal cortical responses to 5-HT [115]. PET imaging studies carried out in depressive patients reported reduced 5-HT_1A_ receptor binding in several brain regions when compared to controls [116-118].

Among the 14 known 5-HT receptors, 5-HT_1A_ receptors are the most abundant in the brain and are either presynaptic or postsynaptic. The downregulation of hippocampal 5-HT_1A_ receptor gene expression and binding in the hippocampus and amygdala arises in response to cortisol hypersecretion in depressive subjects [119]. A relationship between major depression and serotonin transporters, which indicated altered serotonergic availability in depressed patients has been also reported in molecular imaging studies [120, 121]. Accordingly, selective SSRIs such as fluoxetine, paroxetine, sertraline, and escitalopram can enhance brain serotonin levels and are considered the first-line therapies for patients with major depression [122]. Although it has previously been assumed that SSRIs increase the risk for seizures, more recent data suggest that the seizure risk comes from the underlying serotonin deficiency rather than the pharmacologic treatment. In other terms, depression itself is associated with lowering the seizure threshold. Animal models of epilepsy have suggested a potential pathogenic role of 5-HT and NE in epilepsy manifested by decreased serotonergic and noradrenergic activity, as found in the genetically epilepsy-prone rat [123, 124], and the pilocarpine status epilepticus model in the Wistar rat [21]. In these animal models, serotonin dysfunction accompanied also depressive-like behavior [125] suggesting an impact of serotonergic signaling on seizure susceptibility and epilepsy-associated psychiatric comorbidities. Similar 5-HT system alterations have been found in PWE; PET studies showed a reduced 5-HT_1A_ receptor binding in the lateral temporal cortex of patients with TLE and support that these changes are a consequence of down-regulation, internalization, or structural alterations of the receptors [126-128]. The decreased expression of 5-HT_1A_ receptors in the hippocampal or neocortex of patients with MTLE or TLE could lead to high excitability and facilitation of seizure activity [126, 129]. Studies conducted in TLE patients with comorbid depression indicate abnormalities in serotonergic neurotransmission with comparably decreased 5-HT_1A_ receptor binding and 5-HT-transporter activity in specific brain areas involved in both these disorders when compared to TLE patients without depression, independently of the side of the lesion and the degree of hippocampal sclerosis [128, 130-133]. Additionally, a reduced function of the postsynaptic 5-HT1A receptors was shown in an animal model of TLE and concomitant depression, which appears to be caused by a decrease in extracellular serotonin concentrations rather than changes in receptor density [125, 134]. In contrast to postsynaptic receptors, the function of presynaptic 5-HT_1A_ receptors is increased in animals with epilepsy-associated depression, a consequence being strengthened by autoinhibition of 5-HT release and ultimate insufficient neurotransmitter supply into target forebrain areas [125]. Generally, neuroimaging studies including ligand binding to 5-HT_1A_ receptors or the 5-HT transporter indicate a deficit in the serotonergic tone such that both disorders (depression and epilepsy) could coexist, and indeed one pathology could influence the other on this neurotransmission [135]. Enhanced activation of 5-HT_1A_ receptor neurotransmission has been correlated with antidepressant and anticonvulsant effects suggesting an inhibitory role of 5-HT_1A_ receptors in the propagation of seizure activity and depression [136]. Neuroinflammation has been reported to reduce the serotonin output and the upregulation of presynaptic 5-HT_1A_ receptors in raphe nuclei which is also considered one of the potential mechanisms of association between depression and epilepsy [137, 138]. NE has a key role in the pathophysiology of MDD [139] as also supported by the antidepressant effects of monoamine oxidase (MAO) inhibitors and SNRIs [140]. One potential pathogenic mechanism is improved NE sensitivity of α2-adrenoceptors, which can inhibit NE release from the locus coeruleus *via* negative feedback. Indeed, elevated density and enhanced activity of α2-adrenoceptors have been reported in the brain tissues and platelets of patients with major depression [141]. Regarding epilepsy, the noradrenergic system has been strongly involved in the control of seizure activity, especially for patients suffering from partial drug-resistant epilepsy which is frequently triggered within limbic regions [142, 143]. A NE increase seems to be associated with suppression of limbic seizures [143]. NE may play a crucial role in seizure modulation by slowing the stimulation of limbic areas, such as the amygdala and hippocampus [144]. Animal models in which the noradrenergic system, in the locus coeruleus (LC), has been damaged or is genetically compromised generally have a higher susceptibility to experimental seizure (such as accelerated amygdala kindling rate) that can be reduced by repairing noradrenergic activity [142, 145]. The most important antiepileptic effect of NE was evidenced by studies, where NE delayed the onset of amygdala kindling in rats and other models of induced-chemoconvulsants epilepsy [145]. In line with data obtained in these animal models, damage to NE pathways produces increased seizure susceptibility, while the stimulation of LC has a protective effect against epileptic seizures. Additional evidence that NE possesses a strong inhibitory effect on seizure initiation and propagation was obtained by analyzing genetic models of epilepsy in which a decrease in NE has been associated with increased susceptibility to absence epileptic seizures and/or audiogenic seizures and neuronal damage in the limbic regions [146, 147]. The protective effect of NE has been attributed to an antagonizing effect against the formation of an epileptic circuit regulating neuronal changes linked to epileptogenicity. Loss of NE also attenuates the efficacy of a number of anticonvulsant therapies including the ketogenic diet and valproic acid [148, 149]. Finally, NE levels, as well as the density of adrenergic receptors, are reduced following seizures, depending on seizure type or brain area affected. Generally, pharmacological drugs that elevate extracellular NE levels have anticonvulsant actions while NE depletion or administration of adrenergic receptor antagonists increases seizures [142, 148]. In addition to 5-HT and NE neurotransmission deficits, most consistently associated with major depression, DA system dysfunction has also been implicated [150, 151]. Research using neuroimaging, pharmacological, and electrophysiological methods in human and animal models of depression has provided support for the presence of DA dysfunctions [152, 153]. Patients with MDD exhibit low D1 receptor binding potential in bilateral striata with dopaminergic hypofunction [154, 155]. In contrast, increased D2 receptor binding has been detected in several striatal regions [156]. Altered mesolimbic DA system function has been also demonstrated in animal models of depression, such as the chronic mild stress (CMS) model, [157, 158] which is reversed by selective activation of the mesolimbic DA system [159]. Advances in imaging techniques have also provided valuable insights into the contributions of DA to major depression [160]. Studies indicate that dopaminergic neurotransmission is altered in the temporal lobe of patients with TLE. Concerning D1 receptors, there is an increase in protein expression and binding while, lower D2 receptor-induced neurotransmission is detected in patients with drug-refractory epilepsy of longer duration and comorbid psychiatric disorders [161, 162]. These findings seem to be in line with studies in experimental models suggesting a neuroprotective role of DA through the inhibitory control of glutamate neurotransmission and excitotoxicity in epilepsy [163]. Therefore, signaling from D1-like receptors is usually pro-epileptogenic, while D2-like receptor signaling has anti-epileptogenic effects, and the seizures concerning the limbic system appear to be affected by dopaminergic signaling modulation [164].

## 4 ASMS AND DEPRESSION

ASMs can potentially cause psychiatric symptoms, often dose-related, among PWE [17, 165]. These psychiatric side effects are prevalent, particularly in patients with a history of psychiatric conditions or intractable seizures [17]. Furthermore, a recent meta-analysis has linked ASM use with increased suicide risk (FDA, 2008). Of the older ASMs, barbiturates in particular can induce mood changes and behavioral alterations [17]. Also, levetiracetam (LEV), topiramate (TPM), tiagabine (TGB), perampanel (PER), zonisamide (ZNS), and vigabatrin (VGB), are ASMs for which there is strong evidence of frequent psychiatric adverse effects [17]. The risk of depression, as a treatment-emergent adverse event is related to the mechanism of action of ASMs [166]. Conversely, lamotrigine (LTG), valproic acid (VPA), carbamazepine (CBZ), and oxcarbazepine (OXC) are ASMs for which there is strong evidence of antidepressant or mood-stabilizing benefit in PWE. VPA, CBZ, and LTG are also effective as treatments for mania, dysthymic symptoms, and bipolar depression and as mood stabilizers in bipolar and schizoaffective disorders [167-170]. LTG, generally well tolerated, is the only one able to offer symptomatic benefits improving also depressive symptoms associated with bipolar disorder [171]; in addition, LTG has been found to be protective against depressive episodes also in association with LEV when both ASMs were given together [172, 173]. Mixed effects were reported for LEV [174] and TPM [175]. The newer ASMs appear to have better psychological and cognitive profiles. Whilst generally well tolerated, different studies have documented psychiatric and behavioral side effects related to LEV and ZNS [17]; in particular depression, anxiety, and emotional liability have been reported to occur in around 13% and 7% of PWE who used LEV or ZNS respectively [176, 177]. As a result, compared to epileptic patients receiving conventional ASMs, individuals receiving LEV and ZNS treatment may also be at an increased risk of developing psychological illnesses. Brivaracetam, a more potent selective ligand of SV2A than LEV is apparently associated with significantly fewer psychiatric adverse events compared to LEV suggesting better tolerability [178, 179]. Likewise, TPM is associated with a five-fold increased risk of developing depression when rapid titration is performed and therefore should be considered as a contraindication in the use of a rapid titration schedule of this ASM [180]. Furthermore, the effect of rapid titration on the development of depression is amplified by a specific clinical risk factor: the previous history of depression, hippocampal sclerosis, or a history of febrile seizures [173, 181]. Psychiatric adverse events have been identified in the 8% of patients with drug-resistant epilepsy regardless of the mechanism of action of each ASM. This is likely to occur in patients with a predisposition for psychiatric illness, such as those with a past psychiatric history or with a family psychiatric history. Accordingly, to ensure therapeutic success it is mandatory to early identify patients that may be at risk of developing iatrogenic depressive episodes. The rapidity of the titration rate is an important point in order to avoid side effects and not to preclude the opportunity of good seizure control in patients requiring specific ASMs or specific ASMs combinations for their epilepsy syndrome. Even if it is true that some compounds seem to be more frequently associated with behavioral problems than others [182, 183], it is also established that the rapidity of the titration schedule represents one of the most important variables [181].

## 5 CURRENT TREATMENT OF DEPRESSION IN EPILEPSY

Recent clinical practice guidelines suggest a stepped-care model for the management of diagnosed depression in epilepsy. The treatment of depression in both patients with primary mood disorders and individuals with epilepsy is based on an integrated multidisciplinary approach combining pharmacological and psychological interventions [22]. According to recent systematic reviews and meta-analyses, psychological therapy has consistently demonstrated significant effects, by an improvement of comorbid psychiatric symptoms. For patients with mild depression, psychological therapy should be considered as an initial treatment option. It is also advised to be used in conjunction with antidepressants (ADs) for patients with moderate and/or severe depression, as well as for patients who have only partial responses to or issues with adherence to ADs. Patient preference for ADs or psychological therapy and their availability should be considered when deciding between initiating treatment with ADs or psychological therapy [22]. The pharmacological management of depressive disorder is similar to the one provided to patients with primary mood disorders. In general, ADs represent the first-line agents in the treatment of behavioral disorders. For the treatment of major depression, current National and International guidelines, recommend the use of second-generation ADs including SSRIs and SNRIs, which have less toxicity and improved safety compared to the first-generation drugs, which include MAOIs and TCAs. The efficacy of anti-depressants is similar between classes, despite their different mechanisms of action and the variability of the pharmacological properties of the individual drugs.

However, their use in epilepsy has been particularly controversial, since, for a long period, clinicians have believed that they have pro-convulsant properties and therefore represent one of the most frequent obstacles in the treatment of these conditions. Existing evidence on the effectiveness of ADs in treating depressive symptoms associated with epilepsy is still very limited due to a low level of evidence and the poor quality of studies [184]. Rates of response to ADs are highly variable. There are no available comparative data to inform the choice of ADs or classes of drug for efficacy or safety for treating people with epilepsy and depression. The findings obtained from the few uncontrolled clinical studies(four placebo-controlled trials and several open studies) show that many ADs, at therapeutic doses, including TCAs, SSRIs such as sertraline, fluoxetine, and citalopram [185-187], and selective serotonin noradrenaline reuptake inhibitors (venlafaxine and duloxetine) or other ADs like reboxetine and mirtazapine [188, 189] are well tolerated by PWE with no significant seizure aggravation and even these only tend to be associated with an increased risk when used in higher doses [22, 190-192]. Other clinical studies have also reported a favorable response of SSRIs and SNRIs for treatment of depression independently of change in seizure frequency in PWE in open trials, and at lower doses than doses used in primary depressive disorders [193-195]. No influence of citalopram, mirtazapine, and reboxetine on the incidence of seizures has been found in a 30-weeks study in patients with temporal epilepsy and major depression [189].In these studies, not consistent results spanning from low to high efficacy have been reported regarding remission of depressive symptoms [188, 195, 196] although few adverse effects and maintenance of satisfactory seizure control of SSRIs in PWE were observed [22]. Evidence for an at most moderate, but still low, risk (> 0.1% under regular doses) of increased seizures was found for clomipramine, followed by quetiapine, amitriptyline, venlafaxine, citalopram, sertraline, trazodone, mirtazapine, paroxetine, bupropion, and escitalopram. For fluoxetine and duloxetine, the risk seems to be negligible. For the others, mostly newer substances, sufficient evidence was not available [197]. Despite all studies being concordant regarding SSRIs efficacy in the treatment of depression in PWE, in case of inadequate response to an SSRI, switching to venlafaxine has been suggested [22]. Nevertheless, patients with the depressive disorder who are treated with SSRIs and SNRIs are at a higher risk (twofold increased risk) of developing seizures than untreated patients or patients who received TCA treatment with dose-response effects, the period of treatment initiation was associated with the highest risk of seizures [198, 199]. Furthermore, the seizure risk was highest among patients aged between 10 and 24 years and patients with major depression [199]. Therefore, the US Food and Drug Administration (FDA) labeling information for most SSRIs recommends caution in persons with a history of seizures. Animal studies reported both proconvulsant as well as anticonvulsant [200, 201] effects of SSRIs in chronic epileptic animals. In particular, some animal studies determined the ineffectiveness of SSRIs (fluoxetine) in alleviating the depression-like phenotypes associated with epilepsy in the pilocarpine model [21, 202]. Few studies have evaluated whether ADs may influence the processes associated with the development of epilepsy after an insult (*i.e.* epileptogenesis) [12, 203]. A recent large population-based cohort study showed that traumatic brain injury (TBI) was associated with a higher risk of epilepsy in persons who used SSRIs at the time of the TBI compared to those who did not use SSRIs, suggesting a facilitating effect of SSRI exposure on the development of epilepsy after TBI [204]. Also, chronic SSRI antidepressant treatment (both citalopram and fluoxetine) accelerates kindling epileptogenesis [205, 206]. Although the literature is not very well developed, the SSRIs and SNRIs have become the first line of treatment for depressive disorders in PWE, since, in contrast to TCAs, have a favorable pharmacokinetic profile with a limited risk of interactions, and adequate safety at large doses. The new generation Ads seem to be safe when used in combination with ASMs [22]. Anyway, the dosages need to be adjusted according to clinical response, especially when prescribed with inducers such as carbamazepine, barbiturates, and phenytoin, which can reduce the levels of SSRIs by around a quarter. Among other SSRIs, fluoxetine and fluvoxamine are generally not recommended mainly because of the risk of drug-drug interactions and the complex pharmacokinetics. Both of them have an inhibitory effect on the CYP2C9 which may be associated with an increased risk of pharmacokinetic interactions with ASMs metabolized by this enzymatic pathway like phenytoin and partially valproate [207, 208]. Today there is a lack of consent in both preclinical and clinical studies regarding the safety and efficacy of ADs and different patients with treatment-resistant depression (TRD) and epilepsy. Therefore, larger randomized, double-blinded, placebo-controlled clinical trials are necessary to clarify the efficacy and safety of ADs in PWE with different etiologies. Also, it is established that suicide is more frequent in people with epilepsy as compared to the general population [209]. This is even more relevant in the context of depression. Risk assessment and suicide prevention protocols for people with epilepsy are also urgently needed.

### 5.1 Effects of ADs in Epilepsy: Clinical Studies

Data on the treatment of depression in PWE are still limited and rely heavily on individual clinical experience, and only recently general recommendations for health professionals dealing with adults with epilepsy have been proposed [22]. However, a Cochrane meta-analysis on this topic emphasizes the limited data on the efficacy and safety of ADs in PWE and the inadequate quality of available studies [190]. In this Cochrane meta-analysis, only four published randomized controlled trials (RCTs) for ADs in PWE were reported (Table **1**). The first of these RCTs, published over 30 years ago, compared nomifensine, amitriptyline, and placebo in 45 PWE and depression over a period of 12 weeks. Statistical analysis has shown no differences in seizure frequency among these three groups [210]. It's interesting that one patient who had nomifensine treatment was seizure-free. The other two RCTs compared paroxetine to doxepin in 67 people with generalized onset epilepsy and depression, while the other is a controlled study of venlafaxine *versus* placebo in 64 individuals. In these two studies, no changes in seizure frequency were mentioned [211, 212]. The last is a multi-center RCT including 140 patients with focal and/or generalized epilepsy with major depressive disorder comparing sertraline to cognitive behavior therapy (CBT). In this study, depression remitted in more than 50% of PWE within 4 months of initiating treatment with either CBT or sertraline. Furthermore, sertraline treatment was not associated with worsening seizures or suicidality in PWE. Interestingly, the authors affirmed that the remission from depression could be associated with a reduction of generalized seizures. These results should reinforce efforts by clinicians to treat depression in patients who are also suffering the complex clinical and psychosocial effects of epilepsy [213]. Apart from these RCTs, there are several uncontrolled studies on patients with different epileptic diagnoses (Table **1**). In some studies, PWE were monitored for the occurrence of seizures in the months before and then during SSRIs (such as Citalopram, sertraline, and fluoxetine) or SNRIs (such as reboxetine, and milnacipran) antidepressant treatment [185-187, 195, 214] suggest that there is no worsening in seizure frequency and citalopram, fluoxetine, sertraline, and paroxetine appear to be safe as adjunctive therapy to ASMs in PWE and depressive disorders, such as MDD and dysthymia [185, 188]. One study is of interest as it investigated the efficacy and safety of SSRIs in children and adolescents with epilepsy and major depression. In this age group, treatment with sertraline and fluoxetine improved depressive symptoms and ensured the maintenance of satisfactory seizure control with a good safety profile [186].

Kühn *et al*., (2003) have also demonstrated the efficacy and safety of mirtazapine, citalopram, and reboxetine in patients with TLE [189]. Similarly, it has been observed that patients with TLE and depression may also benefit from both CBT and treatment with sertraline and/or citalopram. Furthermore, as documented in this paper, the impact of SSRIs on the number of seizures was superior to that of CBT but was not significantly different [215]. Overall, these studies seem to suggest that SSRIs and/or SNRIs are well tolerated by PWE with no significant seizure exacerbation. These results were also confirmed in a retrospective observational study, in which treatment with SSRIs or SNRIs did not appear to increase seizure frequency in 84 patients with generalized or partial seizures [193]. Three open trials of PWE treated with SSRI such as citalopram or fluoxetine have reported a reduction in seizure frequency by 30% and complete seizure freedom during treatment [188, 194, 196, 214]. The addition of citalopram was tested in epileptic patients without depression and the median seizure frequency was reduced by 56% [196]. Fluoxetine was well-tolerated and reduced seizure frequency in patients with Dravet syndrome, a severe form of epilepsy with a high rate of sudden unexpected death [216]. Other different classes of ADs such as TCAs, SSRIs, SNRI (venlafaxine), α2-antagonist (mirtazapine), and the noradrenaline-dopamine reuptake inhibitor (NDRI) (bupropion) treated depression and also showed positive results on the occasional incidence of seizures reported in PWE [217]. These studies were not powered to show antiseizure efficacy and were not controlled trials and these findings further add to the need for a controlled trial. Thus, an antiepileptic effect of these drugs is yet to be established in PWE. At present, the best evidence for efficacy and safety supports the use of SSRIs and newer ADs, confirming their superiority regarding rates of remission of depressive symptoms, safety profile, and maintenance of satisfactory seizure control. In detail, citalopram was the most frequently used drug followed by sertraline and escitalopram, given their lack (or very limited in the case of sertraline) of pharmacokinetic interactions with AEDs. These drugs induced a decrease in seizure frequency in almost one-third of PWE (27.5%), independently of a change in psychiatric symptoms. Therefore, these drugs may potentially yield a positive effect on seizure frequency in patients with treatment-resistant epilepsy, which needs to be investigated in double-blind, placebo-controlled trials [193]. SSRIs and SNRIs represent the first-line agents for pharmacological treatment [208]. On the other hand, tricyclic or tetracyclic antidepressants and NDRI should be avoided as the first choice. Noradrenergic and specific serotonergic antidepressants (NaSSAs) appear to be safe in epilepsy as well [208]. Some of the various drugs being studied for the treatment of epilepsy have novel modes of action that regulate neuronal targets that may be important in depression. At least three compounds target the serotonergic system, Fenfluramine, Naluzotan, and Buspirone. Fenfluramine has been investigated in Dravet syndrome showing a significant reduction in focal motor seizures [218, 219]. Fenfluramine, a substituted amphetamine, acting primarily as a serotonin releasing agent, has been recently licensed as an add-on therapy for the treatment of seizures associated with Dravet Syndrome [220]. Buspirone and Naluzotan (PRX-00023), 5HT_1A_ receptor agonists, are also well-known anti-anxiety and antidepressant agents. The US National Institute of Neurological Disorders and Stroke (NINDS) has sponsored a randomized, double-blind, placebo-controlled, cross-over Phase II study of Buspirone for the adjunctive treatment of seizures in people with focal epilepsy (NCT01496612). Results are not currently available. Naluzotan is another 5HT_1A_ receptor agonist and is similar to Buspirone but clinical data are limited. NINDS has sponsored a randomized, double-blind, placebo-controlled, cross-over Phase II study of Naluzotan (PRX-00023) for the adjunctive treatment of seizures in people with focal epilepsy (NCT01281956). Results are not currently available [221]. Interestingly, substantial perspectives for future studies come from a case report describing the ability of vortioxetine in the recovery of visual scotomas in a patient with symptomatic occipital lobe epilepsy [222]. Ketamine, a racemic mixture comprising equal parts of (R)-ketamine (or arketamine) and (S)-ketamine (or esketamine), is a non-competitive NMDAR antagonist. Because (S)-ketamine has a higher affinity for NMDAR than (R)-ketamine, esketamine has shown a promising rapid antidepressant effect in patients with treatment-resistant depression in both randomized control clinical trials and real-world observation studies [223, 224, 225] with the recent approval of intranasal S-ketamine by the Food and Drug Administration(FDA) in the United States (FDA, 2019) [226] and by the European Medicines Agency in the European Union [227]. Although ketamine is known to be effective in some cases of status epilepticus [228-230], considering its use for depression, it is unlikely that the drug may have any impact on seizures in PWE.

### 5.2 Effects of ADs in Epilepsy: Preclinical Studies

Different experimental models of epilepsy, both with acquired and genetic etiology, show psychiatric behavioral comorbidities present in PWE. Several studies have investigated the effects of ADs on seizure occurrence in animal models [231] and potential antiepileptic effects of SSRIs have been found in animal models of focal and generalized epilepsy [138, 232] (Table **2**). These positive effects have been found in both acute seizures and chronic epilepsy [231]. Acute Fluoxetine was found to increase the MES threshold while chronic treatment was quite ineffective upon this parameter [233]. Reboxetine, fluoxetine, citalopram, and duloxetine significantly prevented seizure induced by MES and improved survival [234, 235].

Fluoxetine was also effective against PTZ, picrotoxin, 4-aminopyridine (4-AP), and bicuculline-induced seizures [236-239]. In contrast, other studies of the effects of SSRIs in the PTZ model have found inconsistent results [240, 241]. In particular, fluoxetine injected into rats intraperitoneally 30 min before PTZ did not produce any significant difference in latency and intensity of the PTZ-induced seizures [240], while citalopram at low doses decreased and at higher doses increased the seizure susceptibility induced by PTZ [242]. The biphasic dose-dependent properties of SSRIs including citalopram are a well-known phenomenon: low but clinically relevant doses of ADs appear to exert an anticonvulsant effect in a variety of animal seizure models *via* an increase in NE or 5-HT synaptic levels, while proconvulsant activity may be seen at supra-therapeutic doses [243, 244]. However, only a few experimental studies refer to chronic treatment with these drugs. Chronic fluoxetine did not affect the electroconvulsive threshold in mice [245], while proconvulsant action of chronic sertraline and reboxetine was reported [246]. Other evidence has demonstrated that fluoxetine treatment accelerated electrical amygdala kindling epileptogenesis [206]. Acute effects on seizure sensitivity may be related to the innate excitability of seizure circuitry, whereas epileptogenesis involves alterations involving changes to gene expression and the structure and function of neural circuits. Chronic fluoxetine or paroxetine reduces seizure frequency and severity in the pilocarpine seizure model whereas 5-HT depletion increases seizure susceptibility and frequency [200, 247]. Daily administration of fluoxetine for 5 days, but not for 10 days inhibited SRSs in pilocarpine-induced chronic TLE [21, 200]. On the other hand, Mazarati *et al*. found that fluoxetine treatment resulted in a post-treatment decrease in brain excitability suggesting that the anticonvulsant effects of fluoxetine on seizures induced by hippocampal stimulation are observed only after chronic treatment, and persisted for 1 week after the end of fluoxetine administration [21]. Chronic treatment with citalopram or reboxetine at antidepressant doses reduces the frequency of spontaneous seizures in chronic models of epilepsy (focal pilocarpine model of limbic seizures and kainic acid-induced post-status epilepticus), and their anticonvulsant action depends upon the enhancement of endogenous DA and 5-HT transmission and subsequent D_2_ and 5-HT_1A_ receptor stimulation [201, 248]. Reboxetine also exhibits anticonvulsant activity against PTZ-induced seizures [249]. Furthermore, the elevation of 5-HT levels with the SSRIs fluoxetine, citalopram, and sertraline resulted in a dose-dependent seizure frequency reduction in genetically prone rats and non-genetically prone animals, which is coupled with the extracellular serotonergic thalamic concentration [250, 251]), while the 5-HT precursor 5-hydroxytryptophan (5-HTP) has been shown to have anticonvulsant effects when combined with a monoamine oxidase inhibitor (iMAO) [251, 252]. Fluoxetine or citalopram protected against audiogenic seizure in DBA/2 mice reducing also mortality [253]. The pathogenic role of 5-HT has been demonstrated in focal epilepsy models such as electrical stimulation of the hippocampus in cats, bicuculline injection in the deep pre-piriform cortex of rats, and the lithium-pilocarpine model of status epilepticus in Wistar rats [21]. Depletion of 5-HT through pharmacologic or genetic manipulation has been shown to result in a lowering of seizure threshold and 5-HT antagonists reverse the 5-HT mediated anticonvulsant effects of SSRIs such as fluoxetine, as they lower the seizure threshold [254]. In WAG/Rij rats, a genetic model of absence epilepsy and chronic low-grade depression (dysthymia), acute citalopram administration had a mild, non-significant effect, while fluoxetine caused a moderate, significant increase in SWD but a marked increase of SWD was found when the SSRI was administered after pretreatment with a selective 5-HT_2C_ receptor antagonist [255]. Accordingly, chronic fluoxetine treatment in adult epileptic WAG/Rij rats is proabsence, while duloxetine reduces the number and duration of absence seizures. Instead, it was found that early chronic treatment with fluoxetine and duloxetine, had antiepileptogenic effects when administered before seizure onset in WAG/Rij rats and also significantly decreased immobility times during forced swim tests [256, 257]. Acute and chronic administration of venlafaxine, another SNRI, did not influence the maximal electroshock-, PTZ-, and flurothyl-induced convulsions in mice [246], and acute high doses of venlafaxine were proconvulsant in PTZ-induced seizures [258]. However, venlafaxine showed also anticonvulsant action against audiogenic seizures in rats [259] with acute and chronic treatment significantly increasing the electroconvulsive threshold in MES [260]. In rat studies, sertraline provided reductions in the severity of generalized tonic-clonic seizures as well as secondarily generalized seizures [261]. Sertraline prevented tonic-clonic seizures and the epileptiform EEG activity induced by pro-convulsive (4-AP and PTZ) agents [262, 263]; an effect comparable to the clinically established ASM carbamazepine [264, 265]. Vortioxetine, a new AD approved for the treatment of the major depressive disorder (MDD), is a multimodal and selective serotonin reuptake inhibitor (SSRI) antidepressant that combines 5-HT_1A_ receptor agonism with serotonin transporter (SERT) inhibition [266]. Recently vortioxetine was shown to reduce epileptiform activity in the penicillin and PTZ-induced seizure model while aggravating the absence epileptic seizures in the WAG/Rij rats, a genetic animal model of absence epilepsy [267, 268]. Since it is known that 5HT_1A_ receptor agonists have an anticonvulsant and antiepileptic effect [269], while in WAG/Rij rats increases epileptic activity [270], vortioxetine may increase SWD parameters in WAG/Rij rats and reduce penicillin- and PTZ induced seizure through activation of 5-HT_1A_ receptor-mediated hyperpolarization [268]. Vortioxetine also suppressed the number and reduced the severity of seizures improving cognitive deficits in the chronic PTZ-induced kindling rat model [271]. Although it is thought that some ADs increase glutamatergic neurotransmission, which could cause convulsions, SSRIs have been found to inhibit sodium and calcium channels and reduce the potassium-evoked release of glutamate, and enhance GABA receptor activity [272, 273], actions that could all contribute to their anticonvulsant effects [261]. However, at large doses, these drugs may act as convulsants and induce seizures. Some ADs have effects on G-protein coupled K^+^ channels and inhibition of these channels prevents repolarization of action potentials. Others, including SSRIs, may increase the expression of brain-derived neurotrophic factor, a neuronal growth factor that enhances synaptic transmission at excitatory synapses [261]. The pharmacologic effect of SSRIs influences other neurotransmitter systems involved in epileptogenesis and seizure propagation such as cholinergic neurons in the septum and glutamatergic neurons in the hippocampus and forebrain regions, where 5-HT agonists stimulate acetylcholine and inhibit glutamate release, respectively. Furthermore, stimulation of 5-HT_1A_ receptors in thalamic relay neurons results in an increase in GABA release and, consequently, a decrease in excitatory activity necessary for spike-wave discharges (SWDs) in the absence seizure models [274]. Regarding the strong efficacy in models of chronic partial seizures, it could be hypothesized that SSRI treatment may be most effective against focal-onset seizures but the use of models of chronic primary generalized epilepsy would demonstrate the efficacy of SSRIs that was not seen in the acute models. Most animal studies have investigated the effects of a single dose of drug administered and this does not mimic the human condition where the beneficial antidepressant effects occur after several weeks of treatment.

## CONCLUSION

Depression is the most common psychiatric comorbidity of epilepsy in both children and adults, especially in TLE. Depression contributing to a large proportion of the comorbidity in epilepsy can complicate its management and has been associated with reduced adherence to ASMs, increased seizure frequency, and increased risk of suicide. Although the precise processes are still unclear, there is a bidirectional association between epilepsy and depression, and it has been shown in many instances that there are shared pathogenic pathways that make the incidence of one easier when the other is present. In light of these considerations, their early identification and treatment with pharmacological and/or psychological therapy of depression associated with epilepsy are of crucial clinical importance. Clinicians are generally cautious to prescribe ADs to PWE according to several concerns such as aggravation of seizure control, the possibility of interaction with ASMs, and antidepressant effectiveness in resolving depression symptoms; however, at least some ADs and more likely in some specific cases, these drugs may also contribute to seizure control and the overall clinical outcome. Despite the complicated link between epilepsy and depression, the co-administration of SSRIs is regarded, by ILAE clinical practice recent recommendations, to be safe and effective for usage in epilepsy, and SSRIs are considered first-line therapy for the treatment of depression in PWE. In this review, we have highlighted some of the potential implications for effectiveness against seizures of ADs, particularly the effects of these drugs on seizure susceptibility and on the potential interaction with processes that are associated with epilepsy development and progression. Most clinical and preclinical data suggest that the effects of ADs on epilepsy itself are beneficial, indicating that ADs, in particular, the newer generation (*i.e.*, SSRIs and SNRIs) should be considered safe for use in epilepsy. The translational evidence that 5-HT is involved in modulating seizure threshold demonstrates the potential antiseizure effects of SSRIs. PWE who respond poorly to, or are intolerant, to other ADs can be prescribed TCAs with caution since they appear to lower the seizure threshold. However, it is prudent to be cautious about such data. While many studies have examined how SSRIs and SNRIs affect seizures focusing on short-term endpoints, few have examined the underlying processes that may lead to the effects of SSRIs on seizures and in particular on the neurobiological alterations that may influence epileptogenesis. As an example, some studies support an initial antiseizure effect that is not maintained chronically which is in agreement with the fact that ADs impact brain function over time with changes and adaptations inside neural networks. In this review, close and complex pathophysiology between epilepsy and depression has been emphasized, so this relationship should be well considered in future studies due to the effects of ADs as potential therapies in treating epilepsy. Further studies are required to fully appreciate the role of common abnormalities in mediating the bidirectional relationship between depression and epilepsy. Studies on serotonergic receptor alterations (function/expression), particularly of receptors other than the 5HT_1A_ receptor, as well as the effects of ADs on ion channels and the influence of this on seizures and epilepsy are among the primary ones, given the growing body of evidence in the field. Future studies will need to investigate the effects of chronic SSRI exposure, investigating the effects on seizures at clinically relevant time points including prior to seizure onset, at the onset, and the chronic stages, as well as investigating the effects on mediators of epileptogenesis and how these are altered with antidepressant treatment. Last but not least, each AD and each type of epilepsy (different etiologies, different seizure types, *etc.*) represent unique clinical situations; as a result, general concepts may not be applicable in every case, and additional research is needed to pinpoint the unique scenario in order to provide tailored treatment.

## Figures and Tables

**Table 1:** Clinical evidence of antidepressants in patients with epilepsy.

**Study Type**	**Patients**	**Types of Seizures**	**Antidepressant**	**Dose**	**Follow Up (Months)**	**Outcome**	**References**
Prospective controlled trial	100 (49 women, 51 men) PWE and depression (n = 97) or OCD (n = 3). Px age between 6 and 62 years	95 Px with focal epilepsy and 5 with primary generalized epilepsy	Sertraline	Average dose:108 ± 56.9 mg daily (range, 25-200)	12	6 Px had an increase in seizure frequency; the remaining Px not worsened (Px with reduced seizures not mentioned)	[185]
Non-RPCS	36 (19 women, 17 men) PWE and depression. Px age between 6 and 18 years	20 Px with symptomatic focal epilepsy and 16 with cryptogenic focal epilepsy	Sertraline (n = 28), Fluoxetine n =8)	The average dose of sertraline: 111.5 ± 30 mg per day (range, 50-200), Average dose of fluoxetine: 45.7 ± 19 mg per day (range, 20-80).	12	2 Px had an increase in seizure frequency; the remaining Px not worsen (Px with reduced seizures not mentioned)	[186]
Non-RPCS	43 (35 women, 8 men) PWE and HAMD score >15. Px age between 12 and 49 years	12 patients with focal epileptic seizures without secondary generalization, 20 patients with focal seizures with secondary generalization to tonic-clonic seizures and 11 patients with generalized tonic-clonic seizures	Citalopram	Average dose: at the end of the first month was 19.3 ± 2.6 mg daily, whereas at the end of the second month was 22.62 ± 8.3 mg daily	2	No significant seizure worsening	[187]
Non-RPCS	39 PWE and depression. Px mean age of 42.7 years	9 Px with simple focal seizures, 32 with complex focal seizures and focal seizures with secondary generalization	Citalopram	20 mg daily	4	39 Px had improvement in seizure frequency	[188]
Non-RPCS	75 (45 women, 30 men) PWE and HAMD score > 15. Px age between 19 and 68 years	All Px have temporal lobe epilepsy and have had focal and/or generalized seizures.	Mirtazapine (n = 27), Citalopram (n = 33) and Reboxetine (n = 15)	Mirtazapine (32.2 ± 6.8 mg), Citalopram (24.2 ± 8.3 mg) and Reboxetine (6.9 ± 2 mg)	7.5	No significant change in seizure frequency	[189]
Retrospective observational study	84 PWE (18 years or older) and depressive and/or anxiety disorder	Generalized and focal seizures	SSRIs or SNRIs	N/A	6	SSRIs/SNRIs did not appear to worsen seizure frequency. In Px with frequent seizures, SSRIs/ SNRIs were associated with a decrease in seizure frequency	[193]
Non-RPCS	121 (63 women, 56 men) PWE who started taking antidepressants and 300 (166 women, 134 men) PWE not treated with antidepressants	Generalized and focal seizures	1^st^ generation: Tricyclic, tetracyclic antidepressants and sulpiride. 2^nd^ generation: SSRIs and SNRIs. Combinations of different generations	N/A	1, 3, 6 and 12	No significant change in seizure frequency between groups	[195]
Non-RPCS	11 (9 women, 2 men) PWE and depression. Px age between 19 and 67 years	All patients suffered from cryptogenic complex focal seizures, and some had secondary generalization	Citalopram	20 mg daily	8-10	Px had a reduction in seizure frequency by 64.1%	[193]
RCT	45 PWE and depressive disorder. Px age between 18 and 70	Generalized and focal seizures	Nomifensine, Amitriptyline and Placebo	Nomifensine 25 mg Amitriptyline 25 mg	12	No significant change in seizure	[210]
RCT	64 (26 women, 16 men) PWE and depression. Px age between 7 and 60 years	Generalized epilepsy	Venlafaxine	25 mg to 75 mg daily	2	No change in seizure frequency reported	[211]
RCT	67 (35 women, 28 men) PWE and depression. Px age between 14 and 62 years	Generalized epilepsy	Paroxetine (n = 33) and Doxepin (n = 34)	Paroxetine at 10 mg daily and titrated up to 40 mg daily. Doxepin, mean dose 100 mg/day	2	No change in seizure frequency in either the paroxetine or the doxepin treatment groups	[212]
RCT	140 (77 women, 63 men) PWE and depressive disorder were enrolled and randomly assigned to either the sertraline (n = 72) or Cognitive Behavior Therapy (n = 68) groups. Px mean age of 39.6 years	Focal (n = 42), generalized (n = 7) and not definite (n = 23)	Sertraline	50 mg to 200 mg daily	4	Px did not have an increase in seizures or suicidality	[213]
Non-RPCS	17 (8 women, 9 men) PWE and depression. Px age between 18 and 56 years	All Px had complex focal seizures with and without secondary generalization	Fluoxetine	20 mg daily	14	6 Px Seizures free; 11 Px had a reduction in seizure frequency by 30%	[214]
Non-RPCS	15 PWE and depressive disorder. Mean age of Px was 38.6 years	Temporal lobe epilepsy	7 Px received 12 sessions of cognitive behavioral therapy, and 8 Px received an SSRI	Sertraline 200 mg to 400 mg daily or citalopram 20 mg daily	1.5-3	No significant change in seizure	[215]
Case Report	27-year-old woman	Dravet syndrome	Fluoxetine	20 mg daily	12	Marked reduction of seizures	[216]
Case Report	52 years-old women	Generalized epilepsy	Vortioxetine	20 mg daily	2	Recovery of Visual Scotomas (data on seizures frequency not reported)	[222]

**Abbreviations:** HAMD = Hamilton Depression Rating Scale; N/A = Not Available; Non-RPCS = Non-Randomized Prospective Cohort Study; OCD = Obsessive Compulsive Disorder; PWE = Patients With Epilepsy; Px = Patients; RCT = Randomized Controlled Trial; Refs = References; SNRIs = Serotonin-Noradrenaline Reuptake Inhibitors; SSRIs = Selective Serotonin Reuptake Inhibitors.

**Table 2:** Effects of Antidepressant drugs in experimental models of epilepsy.

**Drug**	**Model**	**Dose (mg/kg)**	**Study Protocol**	**Effects on Epilepsy**	**References**
Fluoxetine	MES	15-25; 10	30 min before the test	Increased the electroconvulsive threshold Prevented S-IRA and mortality	[233, 234]
	PTZ	10, 15	30 min prior to PTZ	Increased sz threshold	[238]
	Picrotoxin	20	65 min prior to picrotoxin After 5 days	Increased sz threshold	[236]
	4-AP	10	for 7 days before sz induction	Increased the sz latency time	[239]
	Bicuculline	5-20	1 h prior to bicuculline	Suppression of motor sz	[237]
	Pilocarpine	20	10 consecutive days	Shortened immobility time No change in sz frequency	[21]
	Pilocarpine	20	5 consecutive days after sz induction	Reduced SS	[200]
	GEPRs	15	1h before sound stimulation	Decreased sz intensity and latency	[251]
	GEPRs	30 7-20	1-5 hr before seizure induction Seizure induced every 7 days for 28 days	Reduction sz severity Reduction sz threshold	[250]
	DBA/2	25 15-20	30 min or 72h before sound stimulation	Reduced AGSs incidence and severity Reduced S-IRA after AGSs	[253]
	PTZ	2, 5- 20	30 min prior to PTZ	No significant effects	[240]
	WAG/Rij rat	5.0 10 and 30 day 30 day	Acute injection chronic treatment (7 weeks) ELTT	Moderate increase in SWD Increased SWD Reduced epileptogenesis	[255, 256]
	Amygdala kindling	10	Osmotic mini pump, 30 day administration	Accelerated kindling epileptogenesis	[206]
Citalopram	MES	20	30 min before seizure induction	Prevented S-IRA and improved survival	[234]
	PTZ	0.5 or 1 25-50	30 min prior to PTZ	Biphasic effect on sz threshold	[242]
	Pilocarpine	1 mM	During 4 h of pilocarpine administration	Prevented the generation of sz	[248]
	Kainate	15	For 4 days after the induction of SE	Decreased SS	[201]
	WAG/Rij rat	2.5	Acute injection	No significant effects	[255]
Reboxetine	Kainate	20, 30	Injections for 4 days after the induction of status epilepticus	Decreased SS	[201]
	PTZ	4-15	30 min prior to PTZ	Increased sz threshold	[249]
	MES	0.1-30; 8-16	30 min before seizure induction	Reduced sz severity Enhanced electroconvulsive threshold	[234, 235]
	Flurothyl	20	21 days before seizure induction	Lowered sz threshold	[246]
Paroxetine	Pilocarpine	5	For 4 weeks following SE induction	Reduced SSRs	[247]
Duloxetine	WAG/Rij rat	30 10 and 30	Chronic treatment (7 weeks) ELTT	Reduced SWDs Reduced epileptogenesis	[256]
	MES	10	30 min before seizure induction	Prevented S-IRA and improved survival	[234]
Venlafaxine	MES	12.5, 25	Administration 30 min prior to test Test after 14 days administration	Increased Convulsive threshold	[260]
	Flurothyl	20-40	Administration 30 min prior to test Test after 21days administration	No change Convulsive threshold No change Convulsive threshold	[246]
	PTZ	25-50 75-100	Administration 30 min prior to test Administration 30 min prior to test	Decreased sz severity and latency Increased sz severity and latency	[258]
	Ethanol withdrawal syndrome in rats	20	30 min before ethanol withdrawal testing	Prolonged latency of audiogenic sz	[259]
Sertraline	PTZ	10	14 days prior to test	Increased clonic sz threshold	[262]
	4-AP	2.5, 25 0.75	4 hr before sz induction for 7 days before sz induction	Prevented the increase in EEG amplitude induced	[263, 264]
Vortioxetine	Penicillin	10 1, 5, and 10	After the sz induction 30 min after penicillin	Reduced epileptiform activity Reduced epileptiform activity	[267, 268]
	PTZ	1, 5, and 10	60 min before PTZ	Reduced epileptiform activity	[268]
	WAG/Rij	1, 5, and 10	Before ECOG recordings	No changes for SWDs number and duration	[268]
	PTZ-kindling	5, 10	Before the kindling	Reduced number and seizure severity	[271]

**Abbreviations:** AGSs = Audiogenic Seizures; S-IRA = Seizure-induced Respiratory Arrest; SE = Status Epilepticus; SRSs = Spontaneous Recurrent Seizures; SS = Spontaneous Seizures; SWDs = Spike Wave Discharges; SZ = Seizure.

## LIST OF ABBREVIATIONS

ADs: Antidepressants
ASMs: Antiseizure Medications
CBZ: Carbamazepine
CMS: Chronic Mild Stress
CNS: Central Nervous System
CRP: C-reactive Protein
CSF: Cerebrospinal Fluid
HPA: Hypothalamic Pituitary Adrenal
ILAE: International League against Epilepsy
LC: Locus Coeruleus
OXC: Oxcarbazepine
RCTs: Randomized Controlled Trials
SRSs: Spontaneous Recurrent Seizures
TBI: Traumatic Brain Injury
TLE: Temporal Lobe Epilepsy
TNFα: Tumor Necrosis Factor-alfa
TRD: Treatment-resistant Depression
